# Therapeutic effects of vitamin D supplementation on COVID-19 aggravation: a systematic review and meta-analysis of randomized controlled trials

**DOI:** 10.3389/fphar.2024.1367686

**Published:** 2024-05-27

**Authors:** Yiyuan Yang, Wanli Sun, Fan Yang, Guoxia Zhang, Xinye Li, Shipeng Sun, Yanwei Xing

**Affiliations:** Guang’anmen Hospital, China Academy of Chinese Medical Sciences, Beijing, China

**Keywords:** vitamin D, COVID-19, mortality, ICU admission, mechanical ventilation, meta-analysis

## Abstract

**Background:**

The therapeutic effects of vitamin D supplementation on Coronavirus disease 2019 (COVID-19) aggravation remain controversial and inconclusive. To probe into this contentious issue, we performed the present meta-analysis of randomized controlled trials (RCTs).

**Methods:**

Literature published up to June 2023 was retrieved from Cochrane Library, PubMed, Web of Science and Embase. RCTs assessing mortality, intensive care unit (ICU) admission, mechanical ventilation (MV), length of hospitalization (LOH), and inflammatory markers containing C-reactive protein (CRP), D-dimer, interleukin-6 (IL-6), lactate dehydrogenase (LDH) were included. 19 RCTs were involved in the analysis and were conducted subgroup analyses on the baseline COVID-19 severity and vitamin D administration.

**Results:**

In the severity subgroup, statistically significant effects in moderate to severe group were observed in ICU admission (OR 0.43, 95% CI 0.23, 0.80; *p* = 0.008), MV (OR 0.44, 95% CI 0.27, 0.72; *p* = 0.001) and LOH (SMD –0.49, 95% CI –0.92, −0.06; *p* = 0.027). In the administration subgroup, effects of ICU admission (OR 0.39, 95% CI 0.16, 0.97; *p* = 0.044), MV (OR 0.18, 95% CI 0.07, 0.46; *p* = 0.000) and LOH (SMD –0.50, 95% CI –0.96, −0.04; *p* = 0.034) were more pronounced in patients supplied with multiple-dose vitamin D than single-dose. Although the result of mortality showed no statistically significant effect, it indicated a reduced trend (OR 0.87, 95% CI 0.63, 1.12; *p* > 0.05). The results of inflammatory markers reached no statistical differences.

**Conclusion:**

This meta-analysis revealed that moderate to severe COVID-19 patients supplied with multiple doses of vitamin D were less apt to need ICU admission, mechanical ventilation and have shorter hospital stays.

## 1 Introduction

A downward trend of the Coronavirus disease 2019 (COVID-19) outbreak can be witnessed throughout the world in 2023, but the COVID-19 pandemic has not gone away, with an estimated 767 million confirmed cases and 6.9 million fatalities up to June 2023, according to epidemiological data on the Coronavirus Dashboard of World Health Organization (World Health Organization, 2023). COVID-19, due to the highly infectious SARS-CoV-2 virus, is a respiratory disease of which symptoms range from mild, moderate, and even severe and critical (COVID-19 Treatment Guidelines Panel, 2023). Despite the perception that COVID-19 is primarily a respiratory illness, certain research suggested that the nutritional status of infected individuals may influence the progression of COVID-19 (Li et al., 2021; Silverio et al., 2021). Reportedly, vitamin D insufficiency has come forth as a potential but modifiable risk factor with important implications, and vitamin D’s significance in lowering the severity and incidence of COVID-19 is increasingly established (Im et al., 2020). Observational studies that underwent meta-analyses (Ben-Eltriki et al., 2022; Wang et al., 2022) revealed that COVID-19 patients had noticeably lower vitamin D concentrations in serum and greater odds of SARS-CoV-2 infection, and worse prognosis than healthy controls. Meanwhile, low vitamin D levels are closely tied to rising inflammatory marker levels (Hopefl et al., 2022). In the period of COVID-19, inadequate intake of vitamin D and the status of hypovitaminosis D has developed into public health concern that requires addressing.

During COVID-19, numerous randomized controlled trials (RCTs) have been stimulated to elucidate whether additional intake of vitamin D could prevent COVID-19 aggravation. However, the results were mixed, with some studies claiming statistically significant protective benefits and others reporting null results. Up to this point, published meta-analyses of non-RCTs on this topic account for a larger portion, whereas the number of RCTs in meta-analyses of vitamin D supplementation and COVID-19 is fairly limited. In the meta-analysis with 6 RCTs by Varikasuvu et al. (Varikasuvu et al., 2022), COVID-19 patients supplemented with vitamin D showed fewer rates but no statistically significant differences of ICU admission and mortality, which was consistent with another meta-analysis with 8 RCTs by Kümmel et al. (Kümmel et al., 2022). Intriguingly, the pooled analyses of ICU admission reached statistical significance in the meta-analysis with 9 RCTs by Zaazouee et al. (Zaazouee et al., 2023) and the meta-analysis with 9 RCTs and 14 non-RCTs by Hosseini et al. (Hosseini et al., 2022). Hence, it is urgent to conduct a new meta-analysis of RCTs with a larger sample size to collect emerging evidence and to provide more convincing and valuable information. In addition, evidence supporting the therapeutic effects of vitamin D supplementation with different doses (single dose/multiple doses) on COVID-19 patients with different severity (particularly in mild to moderate/moderate to severe COVID-19 patients) is still not entirely inconclusive. Based on these factors, we aim to collect updated published RCTs and conduct a meta-analysis with larger sample sizes to better illustrate the connection between COVID-19 and vitamin D supplementation. A high focus will be placed on the following investigative questions: 1) in COVID-19 patients with different severity, is vitamin D supplementation a new approach to mitigate the risk of mortality, ICU admission, and mechanical ventilation, and to reduce length of hospitalization and levels of inflammatory markers? 2) in terms of administration, could single-high-dose vitamin D improve the curative effect of COVID-19 in comparison with multiple-dose vitamin D?

## 2 Materials and methods

This meta-analysis was performed and reported in strict accordance with the Preferred Reporting Items for Systematic Reviews and Meta-Analysis (PRISMA) (Page et al., 2021).

### 2.1 Data retrieval and literature search

The research problem was put forward by the principal investigator (YWX). With the database including PubMed, EMBASE, Web of Science, and Cochrane Library, a thorough retrieval of relevant and available literature published up to 19 June 2023, was performed independently by two investigators (YYY and WLS). The search strategy is shown detailedly in Additional Supplementary Table S1. After deduplication, the title and abstract of each retrieved literature were evaluated independently by two co-authors (FY and SPS) to exclude articles that were not related to our study. Any differences were reconciled by consensus or by another two reviewers (GXZ and XYL).

### 2.2 Inclusion and exclusion criteria

To better establish the framework of the research questions and seek evidence, we used the PICOS strategy (patient, intervention, comparison, outcome, study). Finally, we adopted the inclusion as follows: 1) inpatients or outpatients diagnosed with COVID-19, severity at baseline ranged from asymptomatic, mild, moderate, and severe; no limitations on age, gender, or ethnicity; 2) comparing administration of single-dose or multiple-dose vitamin D to placebo or standardized therapy for COVID-19; no limitations on the route of administration, duration of medication, and type of vitamin D; 3) reporting baseline COVID-19 severity, endpoints including ICU admission, mortality, mechanical ventilation, length of hospitalization, and inflammatory markers before and after the intervention; 4) randomized controlled trials published with no restriction in language; exclusion criteria as follows: 1) pregnant or lactating women; 2) taking vitamin D supplementation before/at the recruiting time; 3) types of clinical trials other than RCT such as retrospective studies, observational studies, and pilot protocols.

### 2.3 Study outcomes

Prespecified primary outcomes were the following events encompassing need for ICU admission and MV, mortality in COVID-19 patients. The secondary outcomes were length of hospitalization and changes in the levels of inflammatory markers encompassing CRP, D-dimer, IL-6, and LDH.

### 2.4 Data extraction

Two reviewers (YYY and WLS) independently collected the eligible data from the included RCTs using a pre-designed table. Data comprised the source of study, publication year, location, study design, number of participants, baseline characteristics of participants (mean age, sex, vitamin D status), details between the intervention group and control group, and duration of follow-up. Discrepancies were settled by clear consensus. When the mean values and standard deviations (SD) of the provided outcomes were presented indirectly, we manage to derive the desired value by using an estimation formula based on the given numerical values, such as median, range, sample size, and quartile (Wan et al., 2014; Luo et al., 2016).

### 2.5 Quality assessment and publication bias

Two reviewers (FY and GXZ) independently conducted the quality assessment of the included studies, by the use of Cochrane Collaboration’s bias risk tool. The risk of bias for each domain was categorized as low, high, or unclear by the criteria of the Cochrane Handbook of Systematic Reviews (Higgins et al., 2011). To evaluate potential publication bias, we combined the visual perception of the funnel plot and the values of Egger’s test when the number of included studies was at least 10 (Mavridis and Salanti, 2014). We defined significant publication bias as asymmetric funnel plots or the *p*-value of Egger’s test <0.05. When the funnel plot asymmetry was caused by significant publication bias, we applied the trim and filling method to make the adjustment.

### 2.6 Statistical analysis

In this meta-analysis of RCTs, we performed all our analyses by applying Stata 17.0 (StataCorp, College Station, TX, USA). Treatment effects were summarized as odd ratios (OR) with 95% confidence intervals (CI) for dichotomous outcomes and standard mean differences (SMD) with 95% CI for continuous outcomes. Besides, we used the I^2^ statistic to identify the heterogeneity across studies, and we viewed I^2^ > 50% as statistically significant heterogeneity. When significant statistical heterogeneity was noted, we reported OR/SMD using the random effects model. When I^2^ < 50%, we used the fixed effects model. In conducting all the analyses, we considered the result reaching statistical significance if the *p*-value <0.05. Regarding sensitivity analysis, we undertook a one-study leave-out method for each outcome by eliminating one RCT at a time and by analyzing repeatedly.

## 3 Results

### 3.1 Search results

**FIGURE 1:**
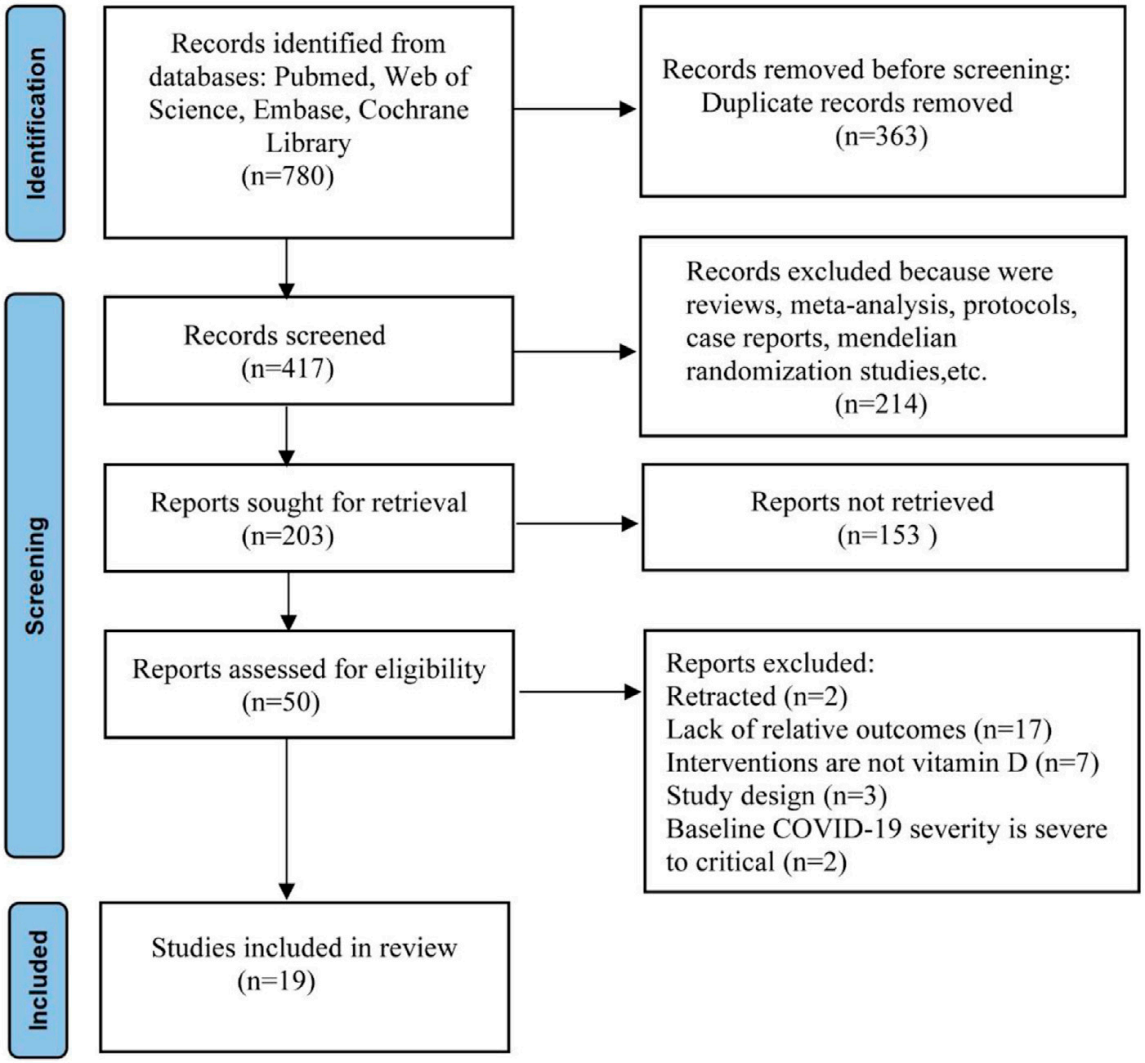
PRISMA flowchart of search strategy.

According to the search strategy, we initially identified 780 articles. After removing duplicates and non-RCTs (reviews, meta-analyses, protocols, case reports, Mendelian randomization studies, etc.), 203 articles remained eventually. Of them, 153 articles were filtered as irrelevant articles after viewing the titles and abstracts; 50 articles were assessed for eligibility; 31 articles were excluded for the below reasons: articles retracted (n = 2), lack of relevant outcomes (n = 17), no vitamin D intervention (n = 7), unexpected study design (n = 3) and baseline COVID-19 severity is severe to critical (n = 2). After undergoing the above screening, 19 RCTs were included in the final meta-analysis. The flowchart diagram of this study selection is displayed in Figure 1.

### 3.2 Study characteristics

The main characteristics of the 19 RCTs (Castillo et al., 2020; Maghbooli et al., 2021; Murai et al., 2021; Sabico et al., 2021; Sánchez-Zuno et al., 2021; Annweiler et al., 2022; Cannata-Andía et al., 2022; Cervero et al., 2022; De Niet et al., 2022; Elamir et al., 2022; Fernandes et al., 2022; Karonova et al., 2022; Mariani et al., 2022; Rastogi et al., 2022; Said et al., 2022; Sarhan et al., 2022; Soliman et al., 2022; Torres et al., 2022; Zurita-Cruz et al., 2022) are summarized in Table 1, with a total of 2,435 participants incorporated. Of these 19 RCTs, there are 7 RCTs making comparisons between the effects of vitamin D and placebo, 7 RCTs between the effects of vitamin D and standard of care, and 5 RCTs between the effects of different dosages of vitamin D supplementation. The majority of studies took *Cholecalciferol* as an intervention, and the majority of participants are vitamin D-deficient and even vitamin D-insufficient at baseline. Vitamin D was dispensed with a single-high dose or multiple doses. The baseline severity of COVID-19 patients varied across the included studies. According to the National Institutes of Health classification (COVID-19 Treatment Guidelines Panel, 2023), COVID-19 infection was categorized into mild disease (defined as non-pneumonia and pneumonia cases, such as mild respiratory symptoms and fever), moderate disease (defined as a lower respiratory disease in clinical evaluation or medical imaging manifestations and the pulse oxygen saturation (SpO_2_) ≥ 94% on indoor air at sea level), severe disease (defined as SpO_2_ < 94% on indoor air at sea level, a ratio of arterial partial pressure of oxygen to fraction of inspired oxygen (PaO_2_/FiO_2_) < 300 mmHg, breathing rate >30 times/min, or pulmonary infiltration >50%), and critical disease (defined as respiratory failure, infectious shock, and/or multiple organ dysfunction). We conducted two subgroup analyses based on the baseline severity of COVID-19 (mild to moderate group, moderate to severe group) and administration of vitamin D (single-dose group, multiple-dose group).

**TABLE 1:** Descriptive summary of included patients and randomized trials characteristics.

Source	Study design and location	COVID-19 severity	Participants					Baseline serum 25OHD deficiency	Treatment Arms				
			Age (year)		Sex (F:M)		Total (N)		No. of patients		No. of patients		Follow-up
										Intervention		Control	
			Intervention	Control	Intervention	Control							
Annweiler et al., 2022 (Annweiler et al., 2022)	France Multicenter, open-label RCT	older adults infected with moderate-severe COVID-19 symptoms	87(IQR:81–92)	89(IQR:83–93)	66:61	82:45	254	Yes	127	High-Dose: Cholecalciferol 400,000 IU at once	127	Standard-Dose: Cholecalciferol 50,000 IU at once	Until 28 days
Cannata-Andía et al., 2022 (Cannata-Andía et al., 2022)	Spain, Argentina, Guatemala and Chile Open label multicenter RCT	patients with moderate-severe COVID-19 disease requiring hospitalization	59(IQR:49–70)	57(IQR:45–67)	93:181	97:172	543	Yes	274	A single oral bolus of 100,000 IU of Cholecalciferol	269	Standard of care	Until discharge or death
Castillo et al., 2020 (Castillo et al., 2020)	Spain open label, double- masked pilot RCT	patients hospitalized with moderate to severe COVID-19 infection	53.14(SD:10.77)	52.77(SD:9.3)	23:27	8:18	76	NA	50	Calcifediol 532 μg at admission, then 266 µg on days 3, 7, 14, 21, and 28	26	Standard of care	Until admission to ICU, discharge or death
Cervero et al., 2022 (Cervero et al., 2022)	Spain Multicenter, single-blinded, prospective pilot RCT	patients diagnosed with moderate-severe COVID-19 pneumonia	67(IQR:58–75)	64(IQR:44–72)	11:30	14:30	85	Yes	41	High-Dose:10,000 IU Cholecalciferol daily for 14 days	44	Moderate-Dose:2000 IU of Cholecalciferol daily for 14 days	Until 14 days
De Niet et al., 2022 (De Niet et al., 2022)	Belgium Double-blind pilot RCT	hospitalized for confirmed SARS-CoV-2 infection with moderate-severe symptoms	63.24(SD:14.46)	68.73(SD:10.97)	8:13	12:10	43	Yes	21	Cholecalciferol 25,000 IU + standard of care at day 1, 2, 3, 4, 8, 15, 22, 29 and 36	22	Placebo + standard of care at day 1, 2, 3, 4, 8, 15, 22, 29 and 36	Until 63 days
Elamir et al., 2022 (Elamir et al., 2022)	Israel Open label RCT	hospitalized patients with mild to moderate COVID-19	69(SD:18)	64(SD:16)	12:13	13:12	50	NA	25	Calcitriol 0.5 µg daily	25	Standard of care	Until 14 days or hospital discharge
Fernandes et al., 2022 (Fernandes et al., 2022)	Brazil Multicenter double blind RCT	patients with moderate to severe COVID-19	55.3(SD:14.2)	55.7 (SD:14.5)	19:58	48:51	200	Yes	101	Single oral dose of 200,000 IU vitamin D3	99	Placebo	Until discharge
Karonova et al., 2022 (Karonova et al., 2022)	Russia open-label, single-center RCT	unvaccinated patients confirmed diagnosis of moderate to severe COVID-19	58(IQR:50–65)	64(IQR:55–70)	NA	NA	110	Yes	56	A bolus of 50,000 IU Cholecalciferol on the 1st and the 8th day, with total dose being 100,000 IU	54	Standard of care	Until 9 days
Maghbooli et al., 2021 (Maghbooli et al., 2021)	Iran, United States of America Pilot multicenter Double-blinded RCT	moderate to severe COVID-19 diagnosed by CT findings compatible with PCR	50(SD:15)	49(SD:13)	22:31	20:33	106	Yes	53	Calcifediol 25 mg orally daily	53	Placebo	Until 60 days
Mariani et al., 2022 (Mariani et al., 2022)	Argentina Multicenter double-blind RCT	SARS-CoV-2 confirmed infection, mild-to-moderate COVID-19	59.8(SD:10.7)	58.3(SD:10.6)	51:64	52:51	218	No	115	A single oral dose of 500,000 IU of Cholecalciferol	103	Placebo	Until discharge
Murai et al., 2021 (Murai et al., 2021)	Brazil Multicenter, double-blind, parallel-group RCT	moderate to severe COVID-19 diagnosed by PCR or by ELISA	56.5(SD:13.8)	56.0(SD:15.0)	49:70	55:63	237	Yes	119	A single, oral dose of 200,000 IU of Vitamin D3	118	Placebo	Until discharge
Rastogi et al., 2022 (Rastogi et al., 2022)	India Randomized, placebo-controlled	asymptomatic or mildly symptomatic SARS-CoV-2 RNA positive individuals	50(IQR:36–51)	47.5(IQR:39.3–49.2)	10:06	10:14	40	Yes	16	Daily 60,000 IU of Cholecalciferol for 7 days	24	Placebo	Until discharge
Sabico et al., 2021 (Sabico et al., 2021)	Saudi Arabia Open label multicenter RCT	patients confirmed SARS-CoV-2 positive diagnosis with mild to moderate symptoms	46.3(SD:15.2)	53.5(SD:12.3)	15:21	20:13	69	Yes	36	5,000 IU Cholecalciferol for 14days	33	Standard of care including 1000IU Cholecalciferol	Until discharge
Said et al., 2022 (Said et al., 2022)	Egypt Open-label RCT	COVID-19 patients with mild to moderate symptoms	50(IQR:20–64)	26(IQR:21–64)	13:17	9:21	60	NA	30	2,000 IU of Vitamin D3 daily	30	Standard of care	Until 14 days
Sánchez-Zuno et al., 2021 (Sánchez-Zuno et al., 2021)	Mexico Open label multicenter RCT	COVID-19 outpatients with mild symptoms	44(IQR:20–71)	43(IQR:21–78)	7:15	6:14	42	Yes	22	10,000 IU daily of Cholecalciferol for 14 days	20	Standard of care	Until 14 days
Sarhan et al., 2022 (Sarhan et al., 2022)	Egypt Prospective RCT	moderate to severe SARS-CoV-2 infected patients	66.1(SD:11.2)	65.7(SD:12.6)	20:38	12:46	116	NA	58	A single high-dose intramuscularly Cholecalciferol 200,000 IU	58	Standard dose of Alfacalcidol 40,000 IU orally	Until discharge
Soliman et al., 2022 (Soliman et al., 2022)	Egypt Prospective RCT	moderate to severe diabetes elderly patients acquired SARS-CoV-2	71.30(SD:4.16)	70.19(SD:4.57)	16:24	6:10	56	Yes	40	Cholecalciferol in a dose of 200,000 units intramuscularly as a single dose	16	Placebo	Until 42 days
Torres et al., 2022 (Torres et al., 2022)	Spain Multicenter, single-blind, prospective RCT	patients with severe COVID-19	67(IQR:58–75)	65.3(IQR:44.0–72.3)	11:30	14:30	85	Yes	41	High-Dose: Cholecalciferol 10,000 IU/day	44	Moderate-Dose: Cholecalciferol 2,000 IU/day	Until 14 days
Zurita-Cruz et al., 2022 (Zurita-Cruz et al., 2022)	Mexico Open-label, single-blind RCT	patients from 1 month to 17 years with moderate to severe COVID-19	10.66(IQR:4.41–14.62)	13.95(IQR:7.35–14.87)	11:09	16:09	45	Yes	20	1,000 IU/day for children <1 years; 2,000 IU/day for children 1–17 years for a minimum of 7 days and a maximum of 14 days	25	Standard of care	Until 7 days or 14 days

Abbreviation:SD, standard deviation; IQR, interquartile range; F = female; M = male; NA, not available.

### 3.3 Risk of bias and quality assessment

**FIGURE 2:**
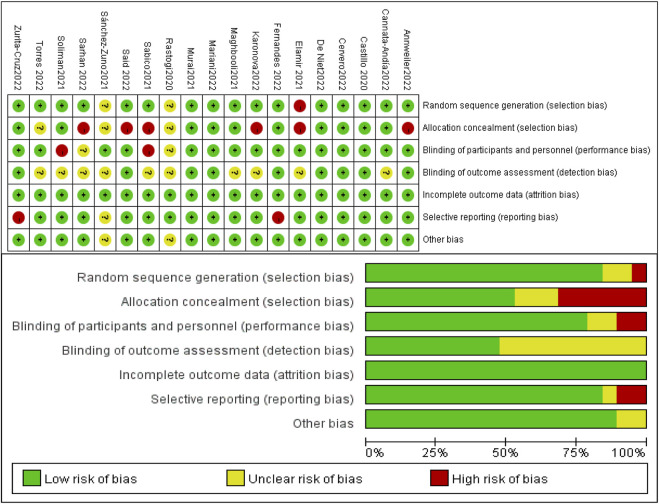
Summary of risk of bias.

The funnel plots and the results of Egger’s test, generated from the data of the included RCTs in the meta-analysis, are listed in Additional Figures 1–3. It was important to note that Egger’s test of ICU admission in the subgroup of severity and administration (*p* = 0.026), mortality in subgroup of administration (*p* = 0.046), showed significant publication bias. However, after further analyses with the trim-and-fill method, the publication bias did not impact the estimates (no trimming performed and no data changed), indicating that publication bias had little effect and verifying the robustness of our results (Duval and Tweedie, 2004). Cochrane Collaboration’s bias risk tool was used to assess the quality of the methodology of included RCTs. The quality assessment of included studies is summarized in Figure 2. Among the 19 RCTs, 47.4% were assessed as high risk of bias, which could be largely attributed to ambiguity blinding setting and possible selective reporting from multiple outcomes. Likewise, 26.3% were assessed as unclear of risk bias, mainly due to problems in the implementation of blinding. 26.3% were assessed as low risk of bias.

### 3.4 Results of meta-analysis

Pooled results calculated by Stata display as shown in Tables 2, 3. Analyses of primary outcomes (ICU admission, mechanical ventilation, mortality) and secondary outcomes (length of hospitalization, inflammatory markers) were assessed, disclosing results as described below.

**TABLE 2:** Results of primary outcomes calculated by Stata.

Primary outcomes			Studies	Participants	OR	95% CI	Z	*p-value*	I^2^ (%)	*P* for heterogeneity
ICU Admission	Severity subgroup	overall effect	13	1,778	0.49	(0.30, 0.79)	2.93	0.003	55.2	0.008
		mild to moderate	3		0.64	(0.32, 1.28)	1.28	0.202	0.0	0.934
		moderate to severe	10		0.43	(0.23, 0.80)	2.67	0.008	66.3	0.002
	Administration subgroup	overall effect	11	1,556	0.48	(0.27, 0.87)	2.44	0.015	59.1	0.006
		single-dose	3		0.67	(0.40, 1.14)	1.48	0.140	0.0	0.546
		multiple-dose	8		0.39	(0.16, 0.97)	2.02	0.044	70.0	0.002
MV	Severity subgroup	overall effect	9	956	0.46	(0.29, 0.72)	3.39	0.001	6.0	0.385
		mild to moderate	2		0.58	(0.19, 1.73)	0.98	0.327	0.0	0.411
		moderate to severe	7		0.44	(0.27, 0.72)	3.27	0.001	20.1	0.276
	Administration subgroup	overall effect	7	734	0.40	(0.25, 0.66)	3.63	0.000	0.0	0.539
		single-dose	3		0.59	(0.32, 1.07)	1.75	0.080	0.0	0.821
		multiple-dose	4		0.18	(0.07, 0.46)	3.58	0.000	0.0	0.948
Mortality	Severity subgroup	overall effect	14	1,983	0.87	(0.63, 1.22)	0.8	0.425	0.0	0.532
		mild to moderate	3		1.09	(0.36, 3.32)	0.15	0.881	34.9	0.215
		moderate to severe	11		0.86	(0.60, 1.21)	0.88	0.377	0.0	0.573
	Administration subgroup	overall effect	11	1,507	1.06	(0.69, 1.64)	0.28	0.779	0.0	0.515
		single-dose	3		1.47	(0.68, 3.17)	0.99	0.322	0.0	0.719
		multiple-dose	8		0.91	(0.54, 1.53)	0.36	0.718	12.1	0.335

Abbreviations: CI , confidence interval; OR, odd ratios; SMD, standard mean difference.

**TABLE 3:** Results of secondary outcomes calculated by Stata.

Secondary outcomes			Studies	Participants	SMD	95% CI	Z	*p-value*	I^2^ (%)	*P* for heterogeneity
LOH (days)	Severity subgroup	overall effect	7	818	−0.33	(-0.66, 0.00)	1.94	0.052	80.3	0.000
		mild to moderate	3		−0.12	(-0.62, 0.39)	0.45	0.652	76.5	0.014
		moderate to severe	4		−0.49	(-0.92, −0.06)	2.21	0.027	78.7	0.003
	Administration subgroup	overall effect	6	702	−0.27	(-0.62, 0.08)	1.51	0.132	78.7	0.000
		single-dose	2		0.07	(-0.35, 0.49)	0.32	0.749	80.9	0.022
		multiple-dose	4		−0.50	(-0.96, −0.04)	2.12	0.034	68.2	0.024
CRP (mg/L)	Severity subgroup	overall effect	10	1,283	0.04	(-0.37, 0.46)	0.21	0.836	91.8	0.000
		mild to moderate	3		0.66	(-0.95, 2.26)	0.80	0.423	95.6	0.000
		moderate to severe	7		−0.16	(-0.54, 0.23)	0.80	0.425	89.0	0.000
	Administration subgroup	overall effect	9	1,167	0.11	(-0.34, 0.55)	0.46	0.645	91.9	0.000
		single-dose	3		0.20	(-0,33, 0.74)	0.75	0.451	89.4	0.000
		multiple-dose	6		0.03	(-0.73, 0.78)	0.07	0.945	93.4	0.000
D-Dimer (ng/mL)	Severity subgroup	overall effect	6	564	0.08	(-0.21, 0.37)	0.52	0.606	59.1	0.032
		mild to moderate	2		0.08	(-0.43, 0.58)	0.29	0.770	39.7	0.198
		moderate to severe	4		0.06	(-0.34, 0.46)	0.31	0.759	71.6	0.014
	Administration subgroup	overall effect	5	448	−0.01	(-0.20, 0.17)	0.12	0.903	42	0.142
		single-dose	1		−0.09	(-0.34, 0.17)	0.68	0.497	—	—
		multiple-dose	4		0.08	(-0.20, 0.35)	0.55	0.582	51.2	0.105
IL-6 (pg/mL)	Severity subgroup	overall effect	4	493	−0.09	(-0.26, 0.09)	0.96	0.337	0.0	0.886
		mild to moderate	1		−0.12	(-0.59, 0.35)	0.49	0.624	—	—
		moderate to severe	3		−0.08	(-0.27, 0.11)	0.84	0.402	0.0	0.731
	Administration subgroup	overall effect	4	493	−0.10	(-0.27, 0.08)	1.05	0.292	0.0	0.907
		single-dose	2		−0.11	(-0.34, 0.11)	0.99	0.322	0.0	0.521
		multiple-dose	2		−0.07	(-0.36, 0.23)	0.44	0.660	0.0	0.781
LDH (U/L)	Severity subgroup	overall effect	5	487	0.12	(-0.06, 0.30)	1.35	0.176	0.0	0.538
		mild to moderate	1		0.01	(-0.46, 0.48)	0.05	0.959	—	—
		moderate to severe	4		0.14	(-0.05, 0.33)	1.44	0.150	0.0	0.411
	Administration subgroup	—	—	—	—	—	—	—	—	—

#### 3.4.1 ICU admission

**FIGURE 3:**
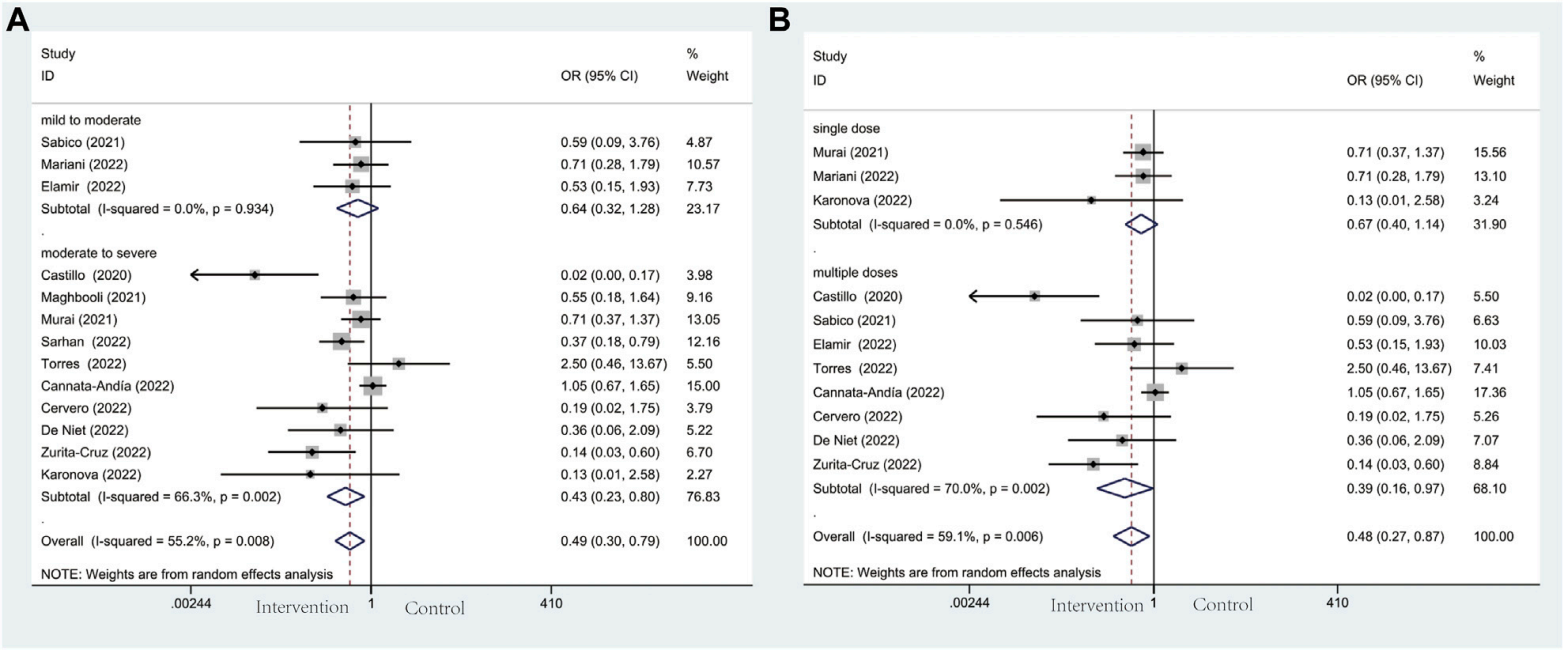
Forest plots of RCT for the association of vitamin D supplementation and ICU admission in the severity subgroup **(A)** and administration subgroup **(B)**.

Thirteen studies including 305 individuals in the vitamin D and the control group reported the events of ICU admission with a total rate of 17.2%. Patients’ need for ICU admission occurred at a rate of 13.6% in the intervention group and 20.9% in the control group, respectively. Patients in the vitamin D group had a decreased probability of COVID-19 progression and a lower frequency of requiring intensive care (OR: 0.49; 95% CI: 0.30, 0.79; *p* = 0.003; I^2^ = 55.2%, *p* = 0.008) (Figure 3A). Notably, in terms of illness severity of COVID-19 patients, comparing moderate to severe with mild to moderate in subgroup analysis, the former showed a lower frequency of ICU admission (OR: 0.43; 95% CI: 0.23, 0.80; *p* = 0.008; I^2^ = 66.3%, *p* = 0.002) (Figure 3A), while the latter showed higher frequency (OR: 0.64; 95% CI: 0.32, 1.28; *p* = 0.202; I^2^ = 0.0%, *p* = 0.934) (Figure 3A). Additionally, subgroup analysis was conducted with eleven studies according to the administration of vitamin D. With the result reaching a statistically significant effect, ICU admission in multiple-dose group (OR: 0.39; 95% CI: 0.16, 0.97; *p* = 0.044; I^2^ = 70.0%, *p* = 0.002) (Figure 3B) is much less frequent than single-dose group (OR: 0.67; 95% CI: 0.40, 1.14; *p* = 0.140; I^2^ = 0.0%, *p* = 0.546) (Figure 3B).

#### 3.4.2 Mechanical ventilation

**FIGURE 4:**
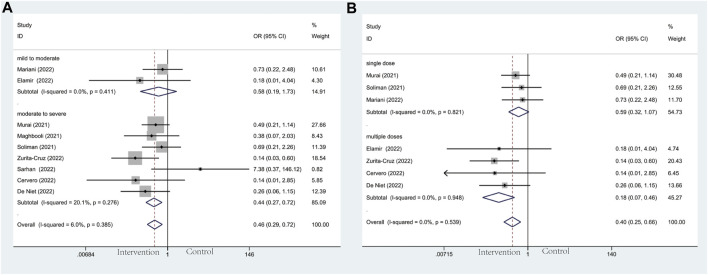
Forest plots of RCT for the association of vitamin D supplementation and mechanical ventilation in the severity subgroup **(A)** and administration subgroup **(B)**.

Modalities of mechanical ventilation can be both invasive and non-invasive. The proportion of need for MV in the vitamin D group and the control groups was 21.1% *versus* 27.6%. Compared with the control group, vitamin D supplementation with lower odds of undergoing mechanical ventilation could be observed (OR: 0.46; 95% CI: 0.29, 0.72; *p* = 0.001; I^2^ = 6.0%, *p* = 0.385) (Figure 4A). Results of subgroup analysis, same as ICU admission, represented more beneficial effects in patients with moderate to severe COVID-19 (OR: 0.44; 95% CI: 0.27, 0.72; *p* = 0.001; I^2^ = 20.1%, *p* = 0.276) (Figure 4A) than in mild to moderate group (OR: 0.58; 95% CI: 0.19, 1.73; *p* = 0.327; I^2^ = 0.0%, *p* = 0.411) (Figure 4A). What’s more, patients who accepted multiple doses of vitamin D (OR: 0.18; 95% CI: 0.07, 0.46; *p* = 0.000; I^2^ = 0.0%, *p* = 0.948) (Figure 4B) are less likely to need MV than those supplied with a single high dose of vitamin D (OR: 0.59; 95% CI: 0.32, 1.07; *p* = 0.080; I^2^ = 0.0%, *p* = 0.821) (Figure 4B).

#### 3.4.3 Mortality

**FIGURE 5:**
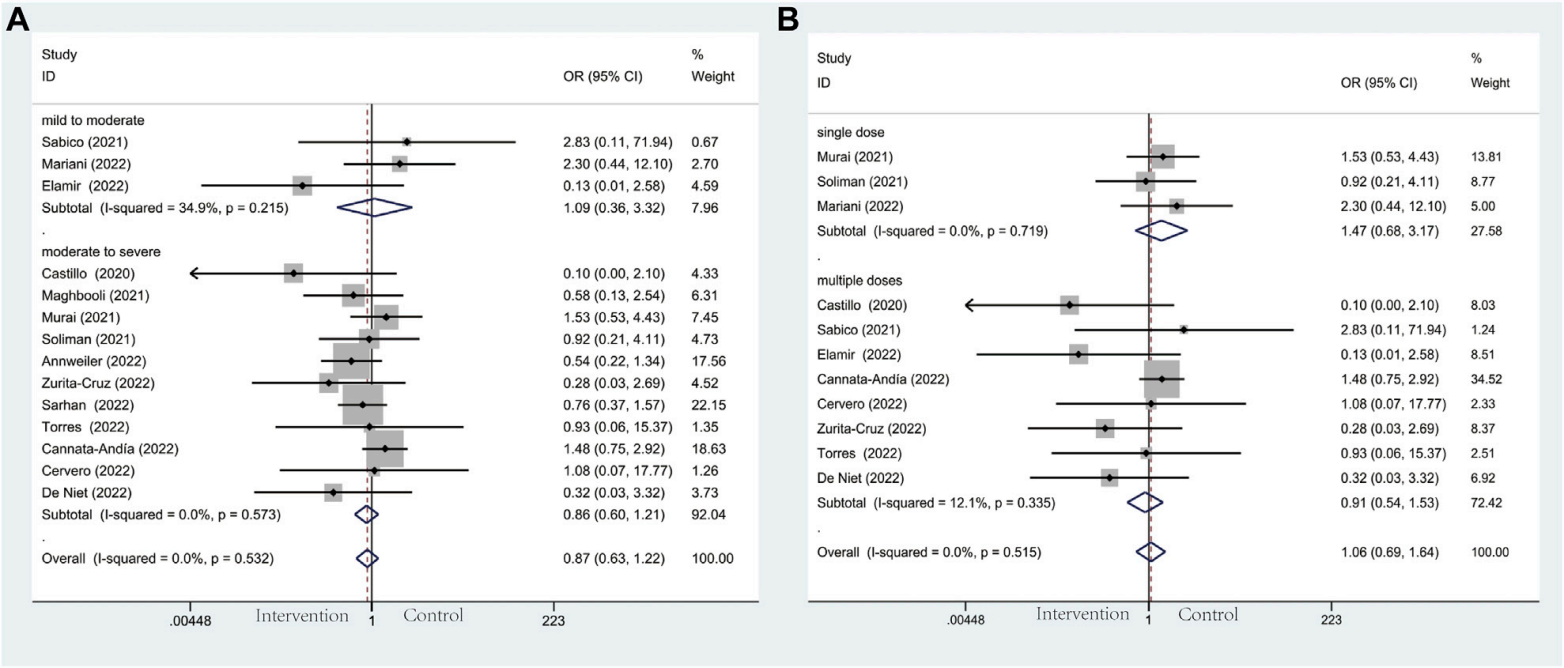
Forest plots of RCT for the association of vitamin D supplementation and mortality in the severity subgroup **(A)** and administration subgroup **(B)**.

For mortality, seventeen trials including 2,175 patients reported this clinical outcome. No deaths occurred at three of these seventeen trials. The death rate in the vitamin D and the control group was 7.6% *versus* 8.4%. Out of line with our hypothesis, the results we performed were not statistically significant in the subgroup analysis of baseline COVID-19 severity (OR: 0.87; 95% CI: 0.63, 1.22; *p* = 0.425; I^2^ = 0.0%, *p* = 0.532) (Figure 5A). However, the results of severity subgroup demonstrated a trend of declining mortality in the intervention group intriguingly. Among these results in detail, no effects were observed in mild to moderate group (OR: 1.09; 95% CI: 0.36, 3.32; *p* = 0.881; I^2^ = 34.9.%, *p* = 0.215) (Figure 5A) and moderate to severe group (OR: 0.86; 95% CI: 0.60, 1.21; *p* = 0.377; I^2^ = 0.0%, *p* = 0.573) (Figure 5A). Similarly, in another subgroup of vitamin D administration, no significant effects were observed in the single-dose group (OR: 1.47; 95% CI: 0.68, 3.17; *p* = 0.322; I^2^ = 0.0%, *p* = 0.719) (Figure 5B) and multiple-dose group (OR: 0.91; 95% CI: 0.54, 1.53; *p* = 0.718; I^2^ = 12.1%, *p* = 0.335) (Figure 5B).

#### 3.4.4 Length of hospitalization

**FIGURE 6:**
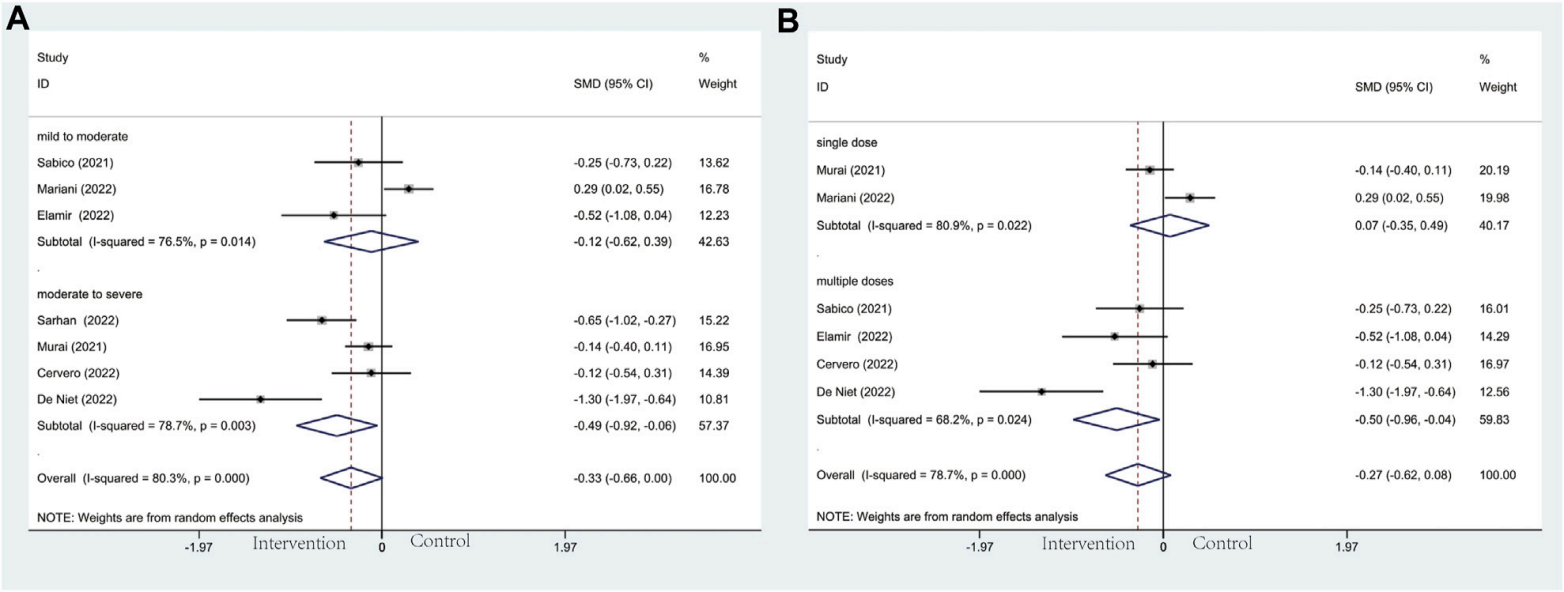
Forest plots of RCT for the association of vitamin D supplementation and length of hospitalization in the severity subgroup **(A)** and administration subgroup **(B)**.

Regarding the length of hospitalization, it was the secondary outcome of this meta-analysis. Based on the observation of the severity subgroup, patients in the moderate to severe group tend to have shorter hospital stays than those in controls (SMD: −0.49; 95% CI: −0.92, −0.06; *p* = 0.027; I^2^ = 78.7%, *p* = 0.003) (Figure 6A), whereas mild to moderate group did not reach statistically significant effect (SMD: −0.12; 95% CI: −0.62, 0.39; *p* = 0.652; I^2^ = 76.5%, *p* = 0.014) (Figure 6A). As for the administration subgroup, a significant difference was observed in the multiple-dose group (SMD: −0.50; 95% CI: −0.96, −0.04; *p* = 0.034; I^2^ = 68.2%, *p* = 0.024) (Figure 6B). However, no noticeable effect was observed in the single-dose group (SMD: 0.07; 95% CI: −0.35, 0.49; *p* = 0.749; I^2^ = 80.9%, *p* = 0.022) (Figure 6B).

#### 3.4.5 Inflammatory markers

**FIGURE 7:**
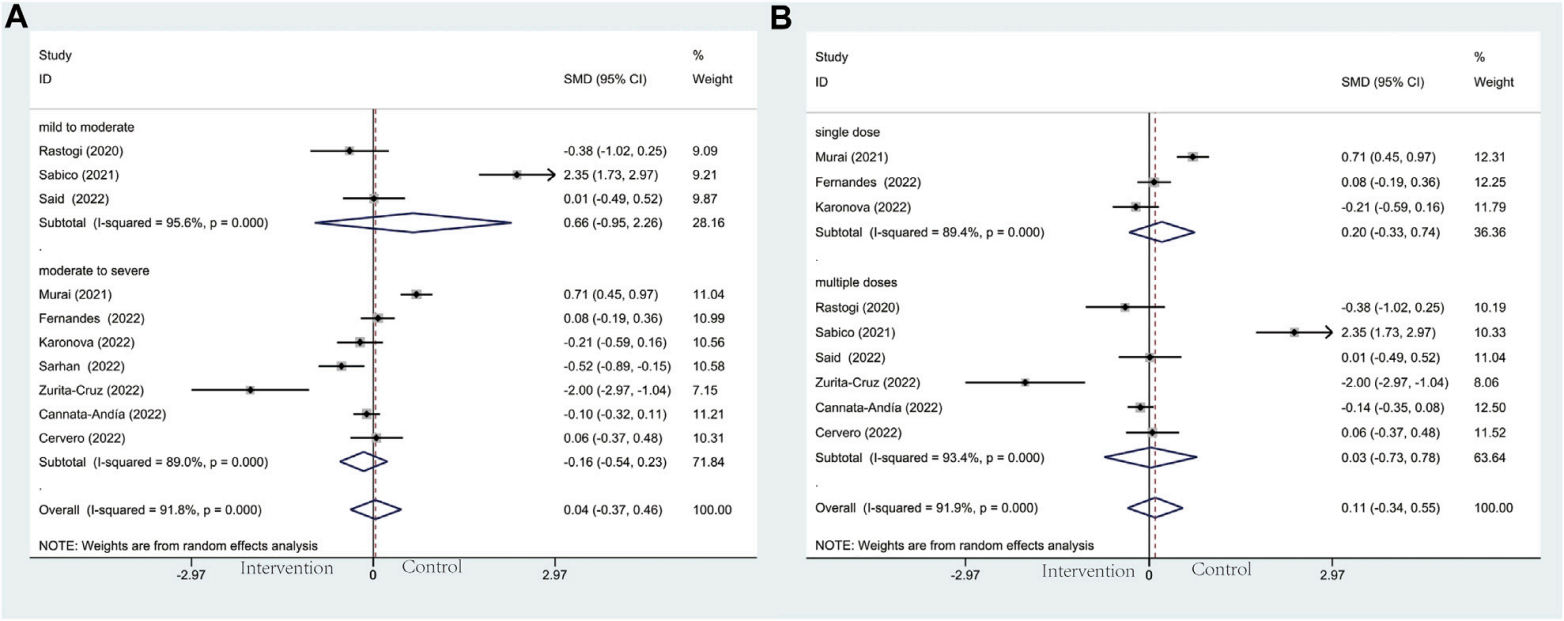
Forest plots of RCT for the association of vitamin D supplementation and CRP levels in the severity subgroup **(A)** and administration subgroup **(B)**.

**FIGURE 8:**
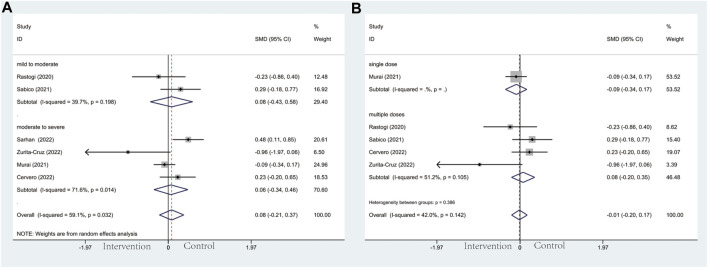
Forest plot of RCT for the association of vitamin D supplementation and D-dimer levels in the severity subgroup **(A)** and administration subgroup **(B)**.

**FIGURE 9:**
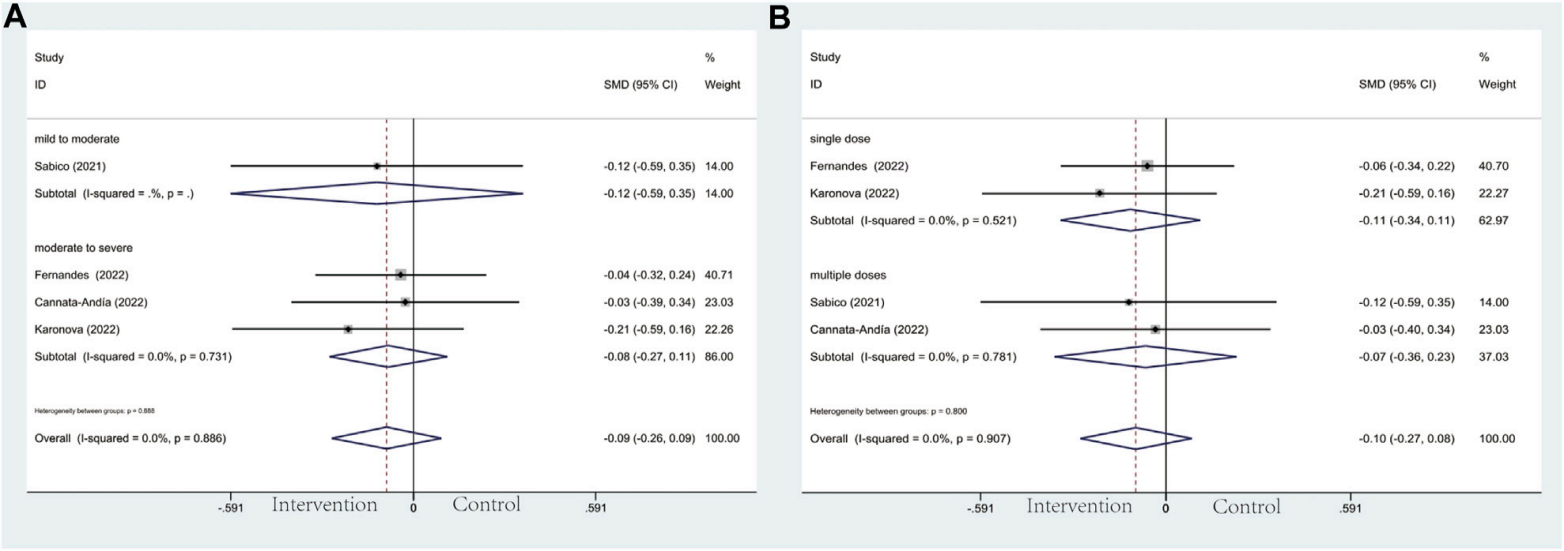
Forest plot of RCT for the association of vitamin D supplementation and IL-6 levels in the severity subgroup **(A)** and administration subgroup **(B)**.

**FIGURE 10:**
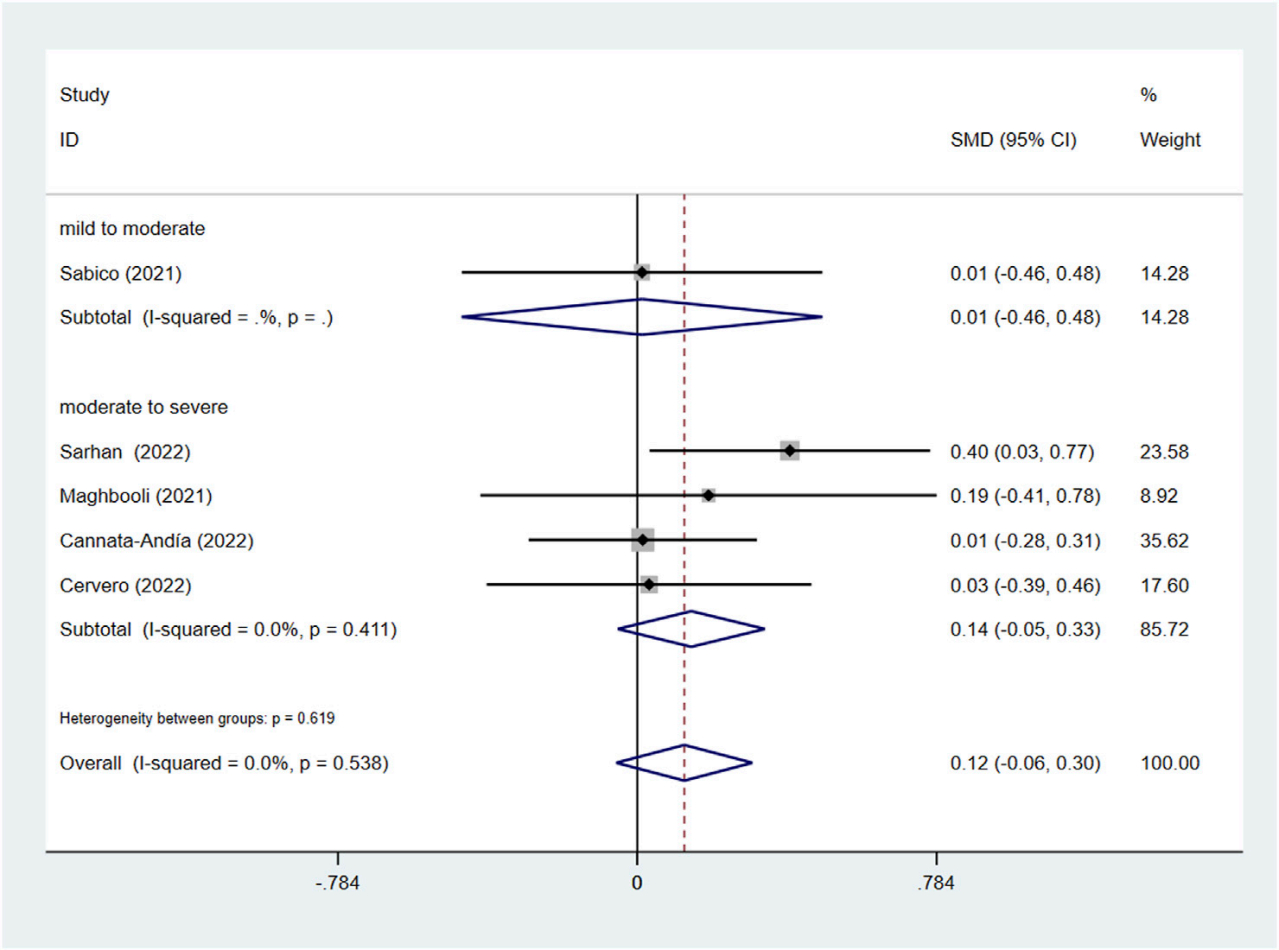
Forest plot of RCT for the association of vitamin D supplementation and LDH levels in the severity subgroup.

In the subgroup of severity, the results did not reach statistically significant differences between the intervention group and control group in CRP (SMD: 0.04; 95% CI: −0.37, 0.46; *p* = 0.836; I^2^ = 91.8%, *p* = 0.000) (Figure 7A), D-dimer (SMD: 0.08; 95% CI: −0.21, 0.37; *p* = 0.606; I^2^ = 59.1%, *p* = 0.032) (Figure 8A), IL-6 (SMD: −0.09; 95% CI: −0.26, 0.09; *p* = 0.337; I^2^ = 0.0%, *p* = 0.886) (Figure 9A), LDH (SMD: 0.12; 95% CI: −0.06, 0.30; *p* = 0.176; I^2^ = 0.0%, *p* = 0.538) (Figure 10). Likewise in the administration subgroup, no statistical difference was observed in CRP (SMD: 0.11; 95% CI: −0.34, 0.55; *p* = 0.645; I^2^ = 91.9%, *p* = 0.000) (Figure 7B), D-dimer (SMD: −0.01; 95% CI: −0.20, 0.17; *p* = 0.903; I^2^ = 42.0%, *p* = 0.142) (Figure 8B), IL-6 (SMD: −0.10; 95% CI: −0.27, 0.08; *p* = 0.292; I^2^ = 0.0%, *p* = 0.907) (Figure 9B). Administration subgroup of LDH failed to meet the condition to conduct further analysis due to inadequate studies.

### 3.5 Sensitivity analysis

We conducted a sensitivity analysis to identify the impact of each trial on the effective index, and ultimately the significant effects of any individual study were unobserved (Additional Supplementary Figures S4–S11).

## 4 Discussion

Centering on the question of whether vitamin D supplementation could diminish the progression of COVID-19, this meta-analysis of 19 RCTs including a distinctly larger sample size than ever before is the first to explore the efficacy of vitamin D in COVID-19 patients with different baseline severity. More convincing than previously published results, the pooled analyses disclosed newfound and statistically significant results that vitamin D supplementation reduced the likelihood of admission to ICU, the need for mechanical ventilation, and the length of hospitalization, especially in moderate to severe COVID-19 patients and those administrated with multiple doses of vitamin D. Nevertheless, the intervention did not significantly transform into decline in mortality or decreased level of inflammatory markers.

Worldwide, a very high prevalence of hypovitaminosis D status has been reported in many countries (van Schoor and Lips, 2017). As an essential nutrient for the human body, vitamin D, of which active metabolite is 1,25(OH)_2_D_3_, plays a participating role in regulating immunoreaction and inflammatory responses to microorganism infections, such as Epstein-Barr Virus, Human Immunodeficiency Virus, Hepatitis B Virus, Human Papilloma Virus, Influenza (Teymoori-Rad et al., 2019), and SARS-CoV-2 (Bilezikian et al., 2020). Malnutrition such as hypocalcemia, hypovitaminosis D in patients has been consistently linked to COVID-19 progression and a worsened prognosis. In a retrospective study conducted by Minasi et al. (Minasi et al., 2023), the relationship between hypocalcemia and adverse clinical outcomes in COVID-19 patients was investigated. The study revealed a significant correlation between serum calcium levels and circulating 25(OH)D. However, the researchers hypothesized that the observed association with the severity of COVID-19 was not directly related to the role of vitamin D in regulating calcium homeostasis. Instead, they suggested that vitamin D might influence the immune response and the production of proinflammatory cytokines. With a direct antiviral effect, vitamin D induces antimicrobial peptides (part of the innate immune system) against enveloped/non-enveloped viruses (Hansdottir et al., 2008; Youssef et al., 2011), and reinforces the barriers made up of cells to help fight off invasive viruses via E-cadherin (Oh et al., 2019). Additionally, vitamin D can suppress cytokine storms which account for acute respiratory distress syndrome (ARDS), by decreasing the production of proinflammatory T-Helper-1 cells (Th-1) and T-Helper-17 cells (Th-17) (Tomaszewska et al., 2022) and increasing the expression of anti-inflammatory cytokines under the regulation of inflammation-related genes (Wöbke et al., 2014). Under most circumstances, the immunopathogenesis of COVID-19 infection involves disruptions in inflammatory mediators. While these disturbances may not directly cause the disease, they contribute to its progression. Some studies claimed that level of Th-17 cells increased in critical COVID-19 patients on account of their body releasing excessive amounts of IL-6 (Xu et al., 2020), indicating the interaction of IL-6 and Th-17 cells in the pathogenesis of COVID-19 with poor prognosis. Most importantly, SARS-CoV-2 infects host cells by utilizing angiotensin-converting enzyme 2 (ACE2) as its receptor (Hoffmann et al., 2020) and leads to the downregulation of ACE2 expression (Lu et al., 2022). Meanwhile, vitamin D can reduce the pulmonary permeability of ARDS by means of mediating the renin-angiotensin system (Malek Mahdavi, 2020). Hence, in COVID-19 patients, downregulation of ACE2 tends to trigger an inflammatory chain reaction and the cytokine storm complicated by ARDS. Vitamin D could be a promising therapeutic approach in patients during COVID-19.

It is noteworthy that vitamin D supplementation shows no significant linkage with mortality and inflammatory markers according to our results, which is consistent with some previous meta-analyses (Hosseini et al., 2022; Kümmel et al., 2022; Varikasuvu et al., 2022; Zaazouee et al., 2023). In accordance with previous evidence, pre-existing vitamin D deficiency predisposes COVID-19 patients to suffer from a worse prognosis. Reportedly, decreased synthesis of vitamin D-binding protein tends to be more common in critical illness, potentially on account of inflammation, injury, disrupted metabolism, and hepatic dysfunction (Jeng et al., 2009; Madden et al., 2015), suggesting that long-term supplemental vitamin D rather than single high-dose vitamin D is preferable. What’s more, a recent study revealed that immune responses of the first line of the human body against viral replication or spread turned out to be slight and delayed, especially in critical COVID-19 patients (Liu et al., 2021). This may help to explain why in the administration subgroup multiple-dose group had more effective results in ICU admission, mechanical ventilation, and length of hospitalization than single-dose group. It is regular long-term vitamin D supplementation that takes a protective effect and offers the human body a relatively suitable circumstance allowing the various beneficial effects to be manifested and reinforced in preventing COVID-19.

Owing to pre-designed study protocols and rigorous screening methods, participants in the majority of previous research were restricted to take vitamin D supplementation before/at the recruiting time, and most of them were at the status of chronic hypovitaminosis D. This may thus be considered as a reason for unimproved mortality and levels of inflammatory markers in COVID-19 patients. At the genetic level, Mendelian randomization studies (Butler-Laporte et al., 2021; Daniel et al., 2022) did not find strong evidence supporting that increasing levels of 25(OH)D protect against COVID-19 severity, but the difference between the finding may be attributed to socioeconomic status and other medical comorbidities.

We acknowledge the limitations of our study. First, participants from single- or multi-centric, open-label, or double-blinded RCTs had several different coexisting diseases and interventions (different dosages, frequencies of administration, and medication duration), leading to the formation of heterogeneity. Second, the absence of reported serum 25(OH)D level after intervention in most studies, limits us to making further evaluations on the efficacy of various administrations and maintenance of optimum dose. Further investigations on vitamin D supplementation maintaining an optimum range of 25(OH)D serum concentration to prevent and alleviate the aggravation of COVID-19 are needed. In the meantime, the most effective and safe method of vitamin D supplementation concerning dosage, route, and duration of administration are all unneglected considerations.

## 5 Conclusion

In conclusion, multiple-dose vitamin D supplementation is closely linked with significantly lower odds of ICU admission, mechanical ventilation, and shorter hospital stays in patients with moderate to severe COVID-19. In the unending era of COVID-19, long-term adherence to a daily intake of vitamin D is recommended to stimulate the immune system and promote anti-inflammatory effects for the purpose of preventing aggravation and poor prognosis after infection with the SARS-CoV-2 virus.

## Data Availability

The original contributions presented in the study are included in the article/Supplementary Material, further inquiries can be directed to the corresponding authors.

## Abbreviations

COVID-19, Coronavirus disease 2019; RCTs, Randomized controlled trials; ICU, Intensive care unit; MV, Mechanical ventilation; LOH, Length of hospitalization; CRP, C-reactive protein; IL-6, Interleukin-6; LDH, Lactate dehydrogenase; PRISMA, Preferred Reporting Items for Systematic Reviews and Meta-Analysis; OR, Odds ratio; SMD, Standardized mean difference; CI, Confidence interval; Th-1 cell, T-Helper-1 cell; Th-17 cell, T-Helper-17 cell.
