# Supplementary material for: Therapeutic effects of vitamin D supplementation on COVID-19 aggravation: a systematic review and meta-analysis of randomized controlled trials
Source: Front Pharmacol. 2024 May 27;15:1367686. doi: 10.3389/fphar.2024.1367686 (PMC11163116; doi:10.3389/fphar.2024.1367686)
Supplement: Supplementary file 1 [file DataSheet1.docx]

**Additional file**

Therapeutic effects of Vitamin D Supplementation on COVID-19 Aggravation: A Systematic Review and Meta-Analysis of Randomized Controlled Trials

Yiyuan Yang^1^, Wanli Sun^1^, Fan Yang^1^, Guoxia Zhang^1^, Xinye Li^1^, Shipeng Sun^1^* and Yanwei Xing^1^*

^1^Guang'anmen Hospital, China Academy of Chinese Medical Sciences, Beijing, China

**Additional content**

**Additional Table.1**. Search of strategy

**Additional Fig.1**. Funnel plots and egger’s test of ICU admission in severity (A) and administration subgroup (B).

**Additional Fig.2**. Funnel plots and egger’s test of mortality in severity (A) and administration subgroup (B).

**Additional Fig.3**. Funnel plots and egger’s test of CRP in severity (A) and administration subgroup (B).

**Additional Fig.4**. Sensitivity analysis of ICU admission in subgroup of severity (A) and administration (B).

**Additional Fig.5**. Sensitivity analysis of mechanical ventilation in subgroup of severity (A) and administration (B).

**Additional Fig.6**. Sensitivity analysis of mortality in subgroup of severity (A) and administration (B).

**Additional Fig.7**. Sensitivity analysis of length of hospitalization in subgroup of severity (A) and administration (B).

**Additional Fig.8**. Sensitivity analysis of CRP in subgroup of severity (A) and administration (B).

**Additional Fig. A.9**. Sensitivity analysis of D-Dimer in subgroup of severity (A) and administration (B).

**Additional Fig.10**. Sensitivity analysis of IL-6 in subgroup of severity (A) and administration (B).

**Additional Fig.11**. Sensitivity analysis of LDH in subgroup of severity.

**Additional Table.1**. Search of strategy

| Pubmed | Embase | Web of Science | Cochrane Library |
| --- | --- | --- | --- |
| (((COVID-19[MeSH Terms]) OR ((((((((((((((((((((((((((((((((((((COVID 19[Title/Abstract]) OR (2019-nCoV Infection[Title/Abstract])) OR (2019 nCoV Infection[Title/Abstract])) OR (2019-nCoV Infections[Title/Abstract])) OR (Infection, 2019-nCoV[Title/Abstract])) OR (SARS-CoV-2 Infection[Title/Abstract])) OR (Infection, SARS-CoV-2[Title/Abstract])) OR (SARS CoV 2 Infection[Title/Abstract])) OR (SARS-CoV-2 Infections[Title/Abstract])) OR (2019 Novel Coronavirus Disease[Title/Abstract])) OR (2019 Novel Coronavirus Infection[Title/Abstract])) OR (COVID-19 Virus Infection[Title/Abstract])) OR (COVID 19 Virus Infection[Title/Abstract])) OR (COVID-19 Virus Infections[Title/Abstract])) OR (Infection, COVID-19 Virus[Title/Abstract])) OR (Virus Infection, COVID-19[Title/Abstract])) OR (COVID19[Title/Abstract])) OR (Coronavirus Disease 2019[Title/Abstract])) OR (Disease 2019, Coronavirus[Title/Abstract])) OR (Coronavirus Disease-19[Title/Abstract])) OR (Coronavirus Disease 19[Title/Abstract])) OR (Severe Acute Respiratory Syndrome Coronavirus 2 Infection[Title/Abstract])) OR (COVID-19 Virus Disease[Title/Abstract])) OR (COVID 19 Virus Disease[Title/Abstract])) OR (COVID-19 Virus Diseases[Title/Abstract])) OR (Disease, COVID-19 Virus[Title/Abstract])) OR (Virus Disease, COVID-19[Title/Abstract])) OR (SARS Coronavirus 2 Infection[Title/Abstract])) OR (2019-nCoV Disease[Title/Abstract])) OR (2019 nCoV Disease[Title/Abstract])) OR (2019-nCoV Diseases[Title/Abstract])) OR (Disease, 2019-nCoV[Title/Abstract])) OR (COVID-19 Pandemic[Title/Abstract])) OR (COVID 19 Pandemic[Title/Abstract])) OR (Pandemic, COVID-19[Title/Abstract])) OR (COVID-19 Pandemics[Title/Abstract]))) AND ((Vitamin D[MeSH Terms]) OR (((((((((((((((((((((((((vitamin d[Title/Abstract]) OR (vit d[Title/Abstract])) OR (Vitamin D 3[Title/Abstract])) OR (Vitamin D3[Title/Abstract])) OR (D3, Vitamin[Title/Abstract])) OR (vit d3[Title/Abstract])) OR (Vitamin D 2[Title/Abstract])) OR (Vitamin D2[Title/Abstract])) OR (D2, Vitamin[Title/Abstract])) OR (vit d2[Title/Abstract])) OR (Ergocalciferols[Title/Abstract])) OR (Ergocalciferol[Title/Abstract])) OR (Calciferol[Title/Abstract])) OR (Calciferols[Title/Abstract])) OR (Cholecalciferol[Title/Abstract])) OR (Cholecalciferols[Title/Abstract])) OR (Calciol[Title/Abstract])) OR (Calcidiol[Title/Abstract])) OR (Calcitriol[Title/Abstract])) OR (calcifediol[Title/Abstract])) OR (25 hydroxyvitamin d[Title/Abstract])) OR (25 hydroxyvitamin d3[Title/Abstract])) OR (25 hydroxycalciferol[Title/Abstract])) OR (1,25 dihydroxyvitamin d[Title/Abstract])) OR (1,25 dihydroxyvitamin d3[Title/Abstract])))) AND (((Randomized controlled trial[Publication Type]) OR (randomized[Title/Abstract])) OR (placebo[Title/Abstract])) | ( 'coronavirus disease 2019'/exp OR 'covid-19':ti,ab OR 'covid 19':ti,ab OR '2019-ncov infection':ti,ab OR '2019 ncov infection':ti,ab OR '2019-ncov infections':ti,ab OR 'infection, 2019-ncov':ti,ab OR 'sars-cov-2 infection':ti,ab OR 'infection, sars-cov-2':ti,ab OR 'sars cov 2 infection':ti,ab OR 'sars-cov-2 infections':ti,ab OR '2019 novel coronavirus disease':ti,ab OR '2019 novel coronavirus infection':ti,ab OR 'covid-19 virus infection':ti,ab OR 'covid 19 virus infection':ti,ab OR 'covid-19 virus infections':ti,ab OR 'infection, covid-19 virus':ti,ab OR 'virus infection, covid-19':ti,ab OR 'covid19':ti,ab OR 'coronavirus disease 2019':ti,ab OR 'disease 2019, coronavirus':ti,ab OR 'coronavirus disease-19':ti,ab OR 'coronavirus disease 19':ti,ab OR 'severe acute respiratory syndrome coronavirus 2 infection':ti,ab OR 'covid-19 virus disease':ti,ab OR 'covid 19 virus disease':ti,ab OR 'covid-19 virus diseases':ti,ab OR 'disease, covid-19 virus':ti,ab OR 'virus disease, covid-19':ti,ab OR 'sars coronavirus 2 infection':ti,ab OR '2019-ncov disease':ti,ab OR '2019 ncov disease':ti,ab OR '2019-ncov diseases':ti,ab OR 'disease, 2019-ncov':ti,ab OR 'covid-19 pandemic':ti,ab OR 'covid 19 pandemic':ti,ab OR 'pandemic, covid-19':ti,ab OR 'covid-19 pandemics':ti,ab) AND ('vitamin d'/exp OR 'vit d':ab,ti OR 'vitamin d 3':ab,ti OR 'vitamin d3':ab,ti OR 'd3':ab,ti OR 'vitamin':ab,ti OR 'vit d3':ab,ti OR 'vitamin d 2':ab,ti OR 'vitamin d2':ab,ti OR 'd2':ab,ti OR 'vit d2':ab,ti OR 'ergocalciferols':ab,ti OR 'ergocalciferol':ab,ti OR 'calciferol':ab,ti OR 'calciferols':ab,ti OR 'cholecalciferol':ab,ti OR 'cholecalciferols':ab,ti OR 'calcidiol':ab,ti OR 'calcitriol':ab,ti OR 'calcifediol':ab,ti OR '25 hydroxyvitamin d':ab,ti OR '25 hydroxyvitamin d3':ab,ti OR '25 hydroxycalciferol':ab,ti OR '1,25 dihydroxyvitamin d':ab,ti OR '1,25 dihydroxyvitamin d3':ab,ti ) AND ('randomized controlled trial'/exp OR 'random':ab,ti OR 'placebo':ab,ti OR 'double-blind':ab,ti ) | TS=(Vitamin D OR vitamin d OR vit d OR Vitamin D 3 OR Vitamin D3 OR D3, Vitamin OR vit d3 OR Vitamin D 2 OR Vitamin D2 OR D2, Vitamin OR vit d2 OR Ergocalciferols OR Ergocalciferol OR Calciferol OR Calciferols OR Cholecalciferol OR Cholecalciferols OR Calcidiol OR Calcitriol OR Calcifediol OR 25 hydroxyvitamin d OR 25 hydroxyvitamin d3 OR 25 hydroxycalciferol OR 1,25 dihydroxyvitamin d OR 1,25 dihydroxyvitamin d3) AND TS=(COVID-19 OR COVID 19 OR 2019-nCoV Infection OR 2019 nCoV Infection OR 2019-nCoV Infections OR Infection, 2019-nCoV OR SARS-CoV-2 Infection OR Infection, SARS-CoV-2 OR SARS CoV 2 Infection OR SARS-CoV-2 Infections OR 2019 Novel Coronavirus Disease OR 2019 Novel Coronavirus Infection OR COVID-19 Virus Infection OR COVID 19 Virus Infection OR COVID-19 Virus Infections OR Infection, COVID-19 Virus OR Virus Infection, COVID-19 OR Coronavirus Disease 2019 OR Disease 2019, Coronavirus OR Coronavirus Disease-19 OR Coronavirus Disease 19 OR Severe Acute Respiratory Syndrome Coronavirus 2 Infection OR COVID-19 Virus Disease OR COVID 19 Virus Disease OR COVID-19 Virus Diseases OR Disease, COVID-19 Virus OR Virus Disease, COVID-19 OR SARS Coronavirus 2 Infection OR 2019-nCoV Disease OR 2019 nCoV Disease OR 2019-nCoV Diseases OR Disease, 2019-nCoV OR COVID-19 Pandemic OR COVID 19 Pandemic OR Pandemic, COVID-19 OR COVID-19 Pandemics) AND TS=(randonmized controlled trial OR randomized OR placebo) | (MeSH descriptor: [COVID 19] explode all trees OR (SARS-CoV-2 Infection):ti,ab,kw OR (2019 nCoV Infection):ti,ab,kw OR (Infection, SARS-CoV-2):ti,ab,kw OR (SARS CoV 2 Infection):ti,ab,kw OR (SARS-CoV-2 Infections):ti,ab,kw OR (2019 Novel Coronavirus Disease):ti,ab,kw OR (2019 Novel Coronavirus Infection):ti,ab,kw OR (COVID-19 Virus Infection):ti,ab,kw OR (COVID 19 Virus Infection):ti,ab,kw OR (COVID-19 Virus Infections):ti,ab,kw OR (Infection, COVID-19 Virus):ti,ab,kw OR (Virus Infection, COVID-19):ti,ab,kw OR (COVID19):ti,ab,kw OR (Coronavirus Disease 2019):ti,ab,kw OR (Disease 2019, Coronavirus):ti,ab,kw OR (Coronavirus Disease-19):ti,ab,kw OR (Coronavirus Disease 19):ti,ab,kw OR (Severe Acute Respiratory Syndrome Coronavirus 2 Infection):ti,ab,kw OR (COVID-19 Virus Disease):ti,ab,kw OR (COVID 19 Virus Disease):ti,ab,kw OR (COVID-19 Virus Diseases):ti,ab,kw OR (Disease, COVID-19 Virus):ti,ab,kw OR (Virus Disease, COVID-19):ti,ab,kw OR (SARS Coronavirus 2 Infection):ti,ab,kw OR (COVID 19 Pandemic):ti,ab,kw OR (Pandemic, COVID-19):ti,ab,kw OR (COVID-19 Pandemics):ti,ab,kw ) AND (MeSH descriptor: [Vitamin D] explode all trees OR (Vitamin D3):ti,ab,kw OR (vit D3):ti,ab,kw OR (Vitamin D2):ti,ab,kw OR (vit d2):ti,ab,kw OR (Ergocalciferols):ti,ab OR (Ergocalciferol):ti,ab,kw OR (Calciferol):ti,ab,kw OR (Calciferols):ti,ab,kw OR (Cholecalciferol):ti,ab,kw OR (Cholecalciferols):ti,ab,kw OR (Calcidiol):ti,ab,kw OR (Calcitriol):ti,ab,kw OR (Calciferols):ti,ab,kw OR (Calcifediol):ti,ab,kw OR (25 hydroxyvitamin d):ti,ab,kw OR (25 hydroxyvitamin d3):ti,ab,kw OR (25 hydroxycalciferol):ti,ab,kw OR (1,25 dihydroxyvitamin d):ti,ab,kw OR (1,25 dihydroxyvitamin d3):ti,ab,kw  ) |

**
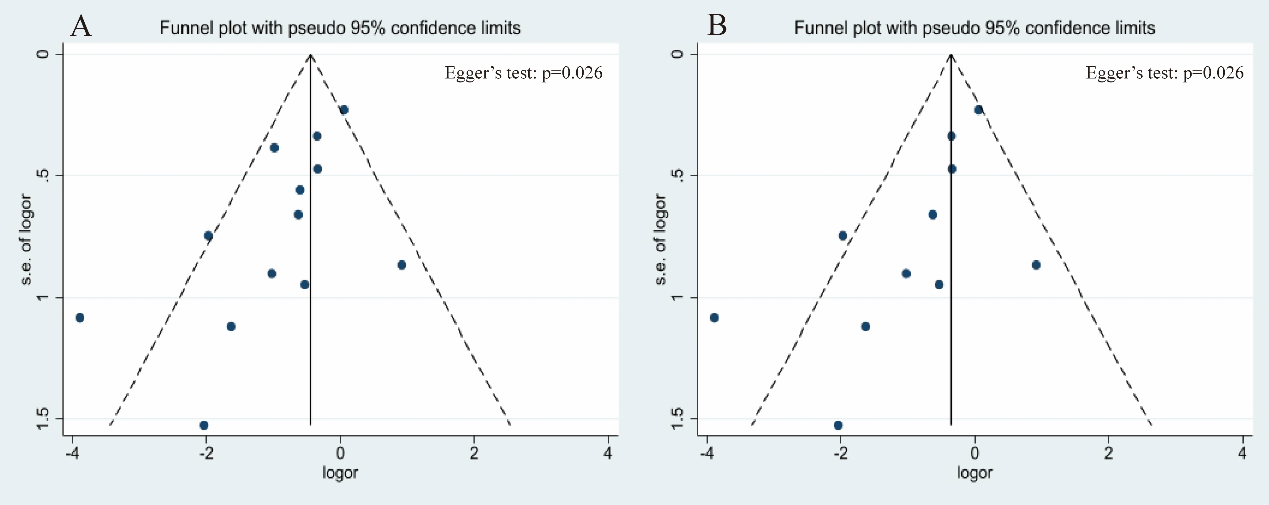
** **Additional Fig.1.** Funnel plots and egger’s test of ICU admission in severity (A) and administration subgroup (B).

**
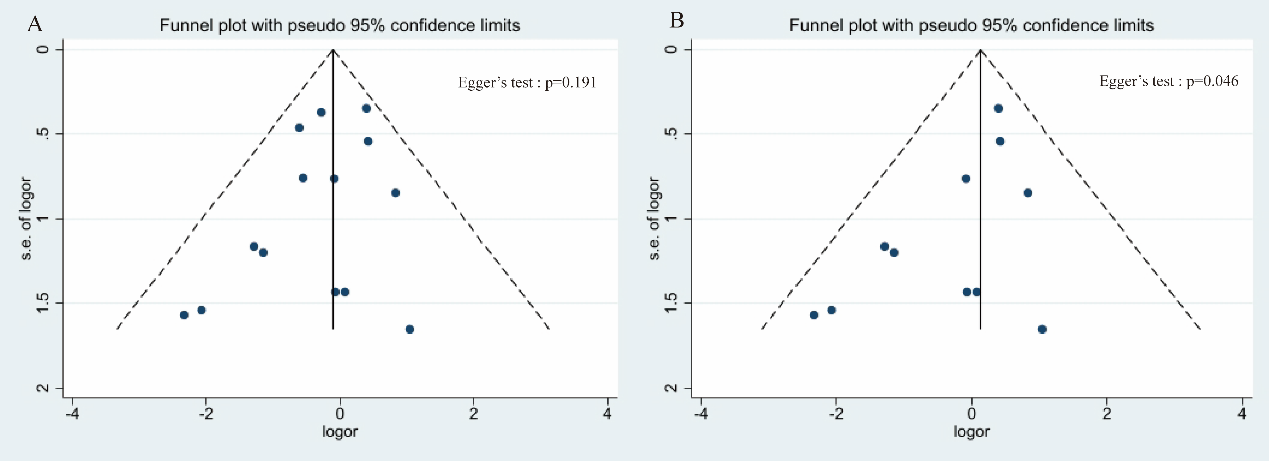
 Additional Fig.2**. Funnel plots and egger’s test of mortality in severity (A) and administration subgroup (B).

**
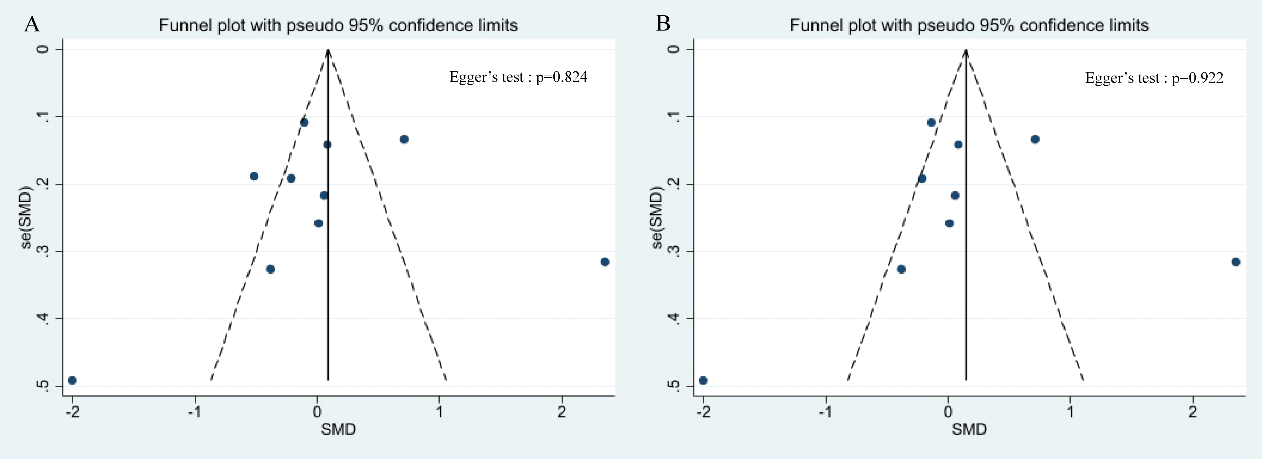
 Additional Fig.3**. Funnel plots and egger’s test of CRP in severity (A) and administration subgroup (B).

**
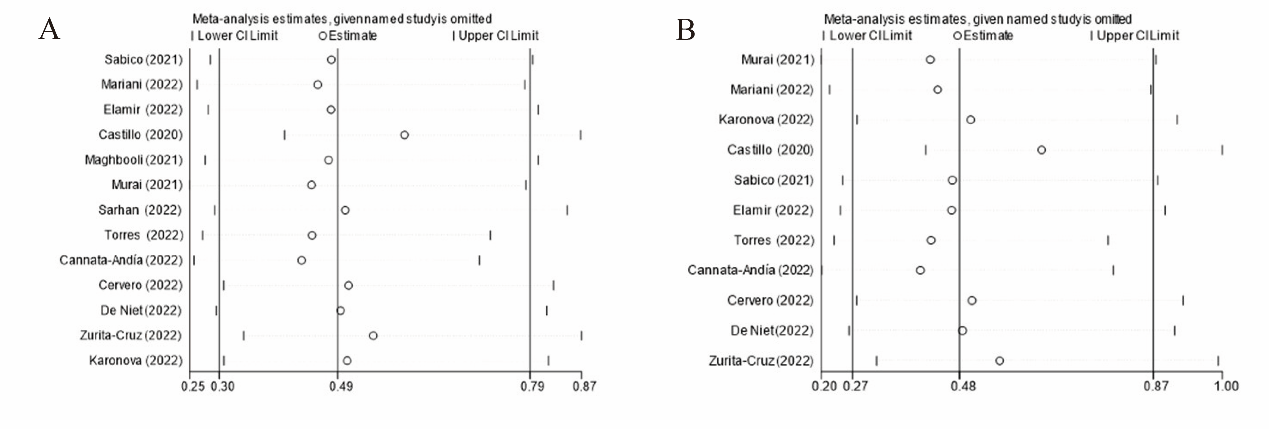
** **Additional Fig.4**. Sensitivity analysis of ICU admission in subgroup of severity (A) and administration (B).

**
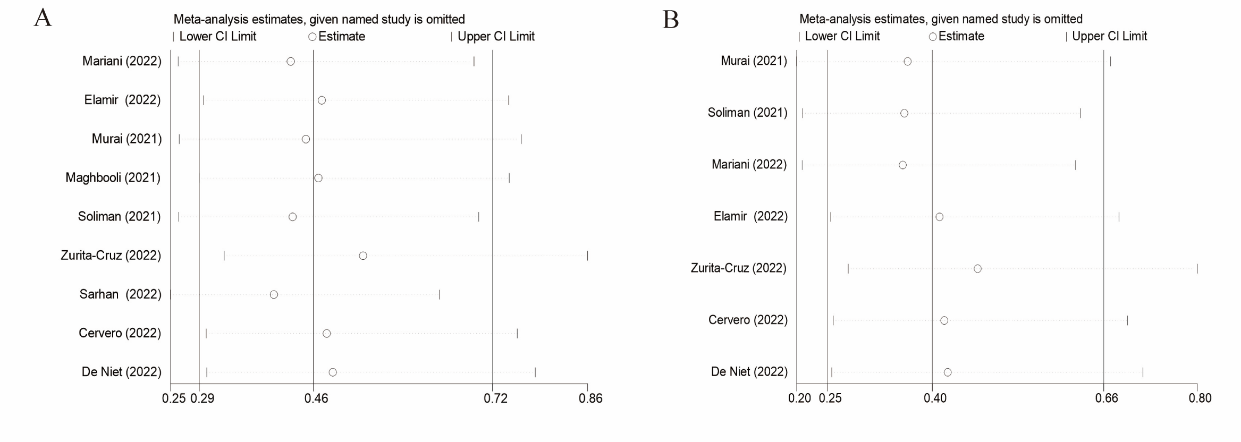
** **Additional Fig.5**. Sensitivity analysis of mechanical ventilation in subgroup of severity (A) and administration (B).

**
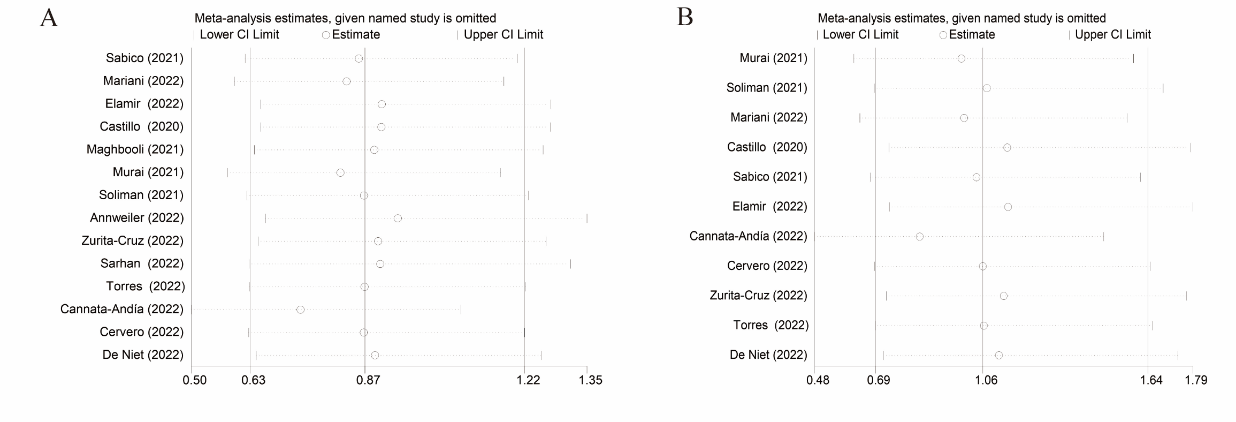
** **Additional Fig.6**. Sensitivity analysis of mortality in subgroup of severity (A) and administration (B).

**
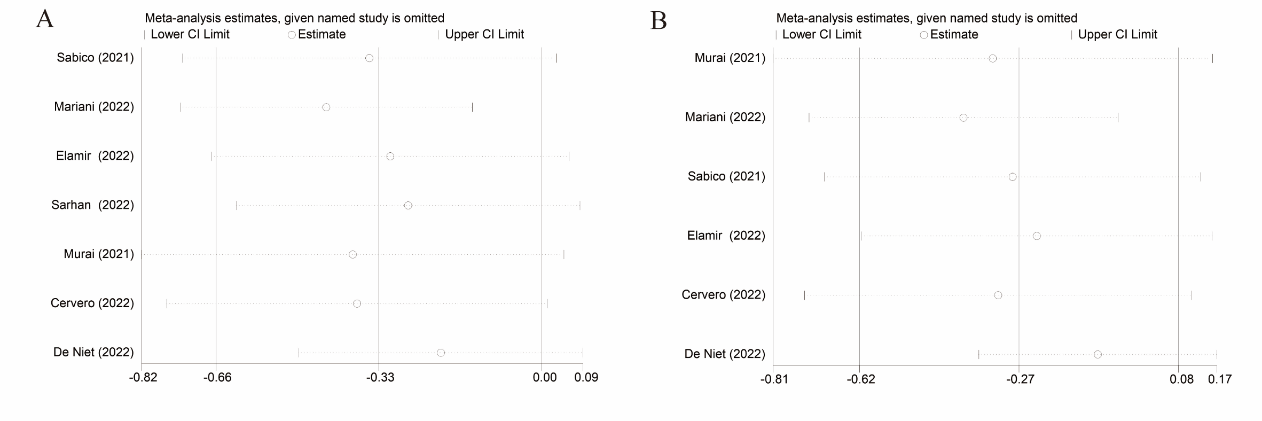
** **Additional Fig.7**. Sensitivity analysis of length of hospitalization in subgroup of severity (A) and administration (B).

**
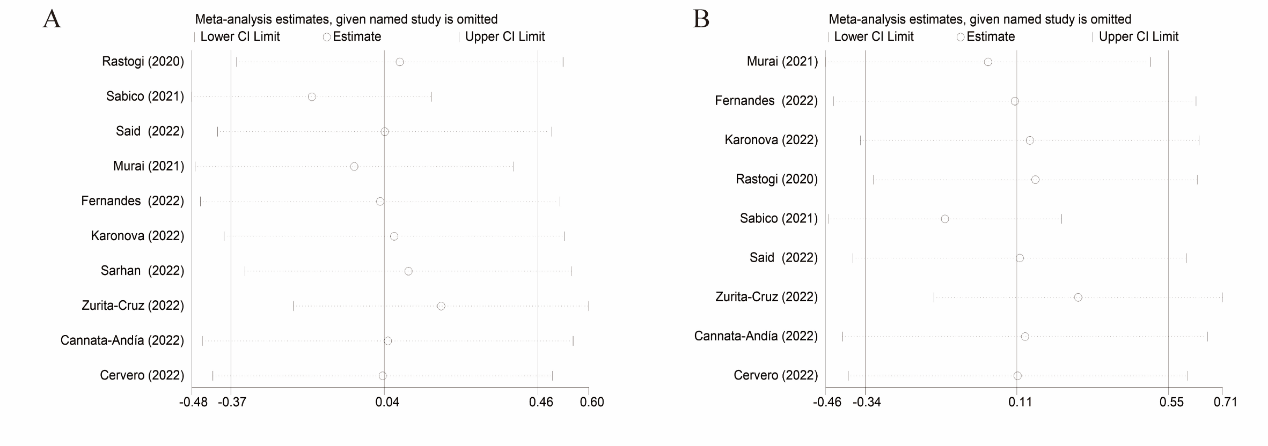
** **Additional Fig.8**. Sensitivity analysis of CRP in subgroup of severity (A) and administration (B).

**
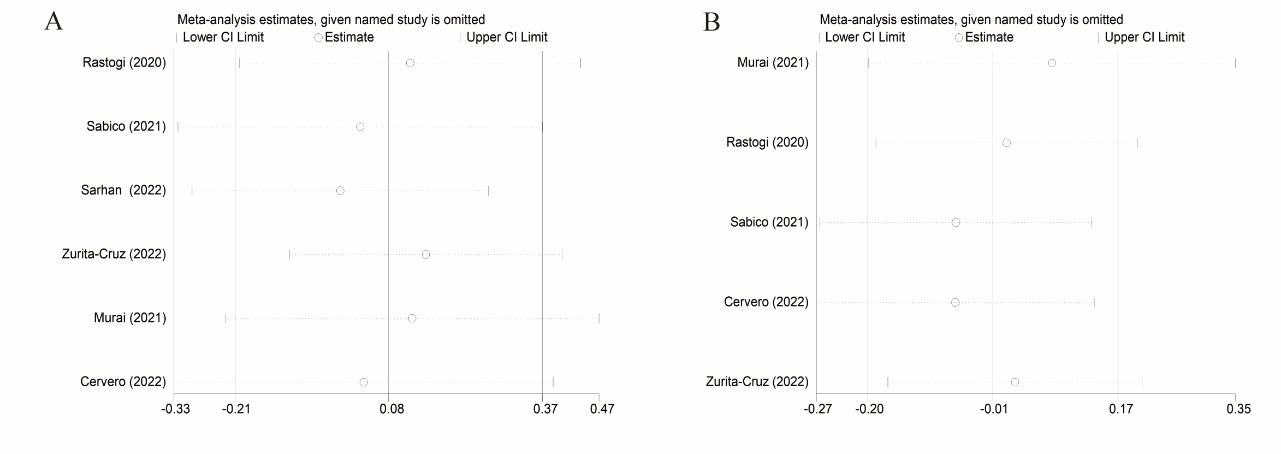
**

**Additional Fig.9**. Sensitivity analysis of D-Dimer in subgroup of severity (A) and administration (B).

**
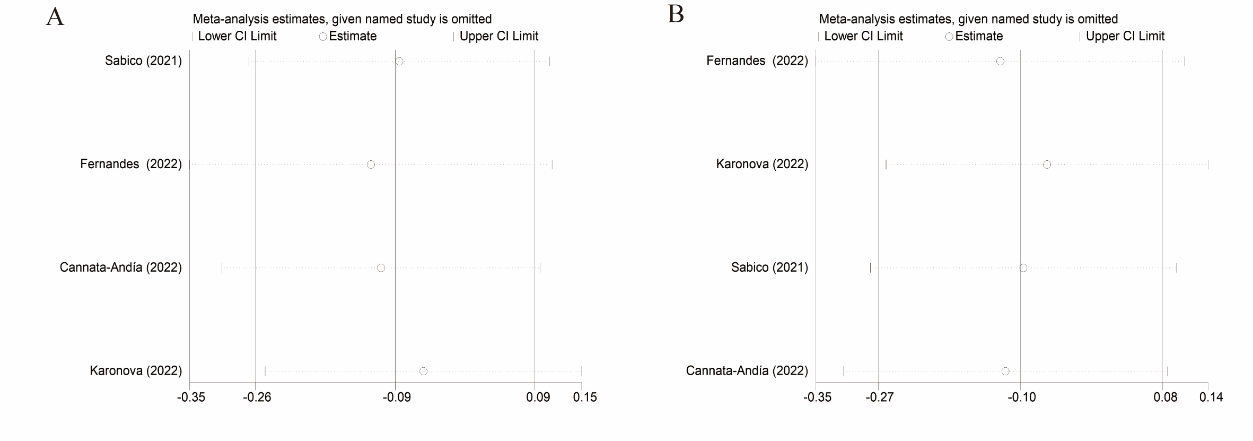
**

**Additional Fig.10**. Sensitivity analysis of IL-6 in subgroup of severity (A) and administration (B).

**
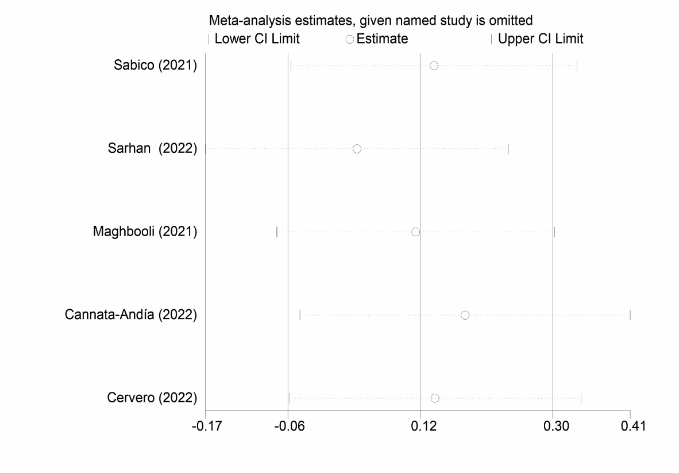
**

**Additional Fig.11**. Sensitivity analysis of LDH in subgroup of severity.
